# Comparison study of population-based methods for non-invasive fetal electrocardiography extraction

**DOI:** 10.3389/fmed.2026.1832787

**Published:** 2026-06-18

**Authors:** Akshaya Raj, Jindrich Brablik, Radana Vilimkova Kahankova, Rene Jaros, Katerina Barnova, Martina Litschmannova, Vaclav Snasel, Seyedali Mirjalili, Radek Martinek

**Affiliations:** 1 Department of Cybernetics and Biomedical Engineering, Faculty of Electrical Engineering and Computer Science, VSB–Technical University of Ostrava, Ostrava, Czechia; 2 Hospital AGEL Trinec-Podlesi, Trinec, Czechia; 3 Centre for Artificial Intelligence Research and Optimization, Torrens University Australia, Melbourne, VIC, Australia

**Keywords:** fetal monitoring, non-invasive fetal electrocardiogram, population-based algorithms, sequential analysis, stochastic algorithms

## Abstract

This paper presents a comparative analysis of five popular population-based algorithms in the field of non-invasive fetal electrocardiogram (NI-fECG) extraction: (1) artificial bee colony (ABC), (2) gray wolf optimization (GWO), (3) moth flame optimization (MFO), (4) particle swarm optimization (PSO), and (5) whale optimization algorithm (WOA). The five optimization algorithms are used along with sequential analysis (SA) to extract the fetal electrocardiogram (fECG) signal that is present along with other signals in the abdominal electrocardiogram. The most prominent one is the mECG signal, which also overlaps with fECG in time and frequency domains making its extraction challenging. The extraction systems were tested on two available datasets (Labor and Pregnancy) and their efficiency was evaluated using the accuracy of the R-peak detection. The algorithms are stochastic in nature; therefore, the experiments were conducted 30 times independently to observe any potential instability. The results indicate that extraction systems with GWO, MFO, PSO, and WOA demonstrated similar performance in the task, showing comparable extraction accuracy. However, the ABC-based system performed poorly and exhibited instability. It is suggested that further investigation into hybrid approaches and further modifications to the ABC algorithm could potentially enhance its extraction accuracy.

## 1 Introduction

Deterministic methods are considered classical algorithms for optimization. A group of deterministic algorithms, which uses gradient information is known as gradient-based. A well-known gradient-based algorithm is the Newton-Raphson algorithm (1), which utilizes function values and their derivatives. It works well for unimodal problems, however, if there is a discontinuity in the objective function, it does not work as well. In case of such occurrences, a non-gradient or a gradient-free algorithm is preferred. These algorithms use only the function values and not the derivatives. Examples of gradient-free algorithms include Hooke-Jeeves pattern search (2) and Nelder-Mead downhill simplex (3).

Another category of optimization algorithms is stochastic. In general, there are two types: heuristic and metaheuristic, where heuristic means “to find or discover by trial and error.” With such algorithms, quality solutions to a difficult optimization problem can be found within a certain period, but there is no guarantee that the solution will be optimal. These algorithms are good when one does not necessarily require an optimal solution, but rather a good solution that is attainable. Further development on the heuristic algorithm led to a new set of algorithms known as meta-heuristic algorithms. Here the term meta means “higher level,” and these algorithms perform better than simple heuristics. These algorithms use certain trade-offs of randomization and local search. This allows the algorithms to move away from local optima to search on a global scale. Therefore, meta-heuristic algorithms tend to be more suitable for global optimization (4, 5).

With all that said, the field of optimization has a wealth of algorithms that only advance with time. Yet, we are left with a question: what is the best algorithm for a well-established problem? A simple answer to such a simple question is that there is no universal algorithm that suits all. One of the reasons for that is the complexity and diversity of the real-world problems that we face today. Not all optimization problems are made alike, and therefore, there is no single method that can cope with all the problems, no matter how well established. This is well stated by the theorem called the no-free-lunch theorem (6). This has allowed various researchers to come up with an optimization algorithm suitable for a particular optimization problem (7–10).

This paper focuses on the optimization problem in the area of *fetal monitoring*, a crucial component of prenatal care that enables healthcare providers to assess fetal well-being. One of the emerging techniques of fetal monitoring is non-invasive fetal electrocardiography (NI-fECG). The method is based on capturing the electrical activity of the fetal heart (fetal electrocardiogram, fECG) along with other signals, using electrodes placed on the mother's abdomen. Compared to the most prevalent technique in the clinical practice, cardiotocography (CTG), NI-fECG offers an analysis of additional features besides fetal heart rate (such as QT and ST segment analysis), allowing more accurate detection of fetal distress (11, 12). Additionally, NI-fECG is a completely passive method and thus does not require any energy to be transmitted into a pregnant woman's body, which makes it suitable for long-term continual monitoring, contrary to the CTG (13). However, NI-fECG monitoring does have its challenges, primarily the presence of various types of noise in the environment but also those produced by the maternal body, such as mECG. These signals can interfere with the fECG and make it difficult to extract and determine the fetal heart rate (fHR) accurately. As a result, specialized filtering and signal processing algorithms are required to extract the fetal signal from the composite abdominal mixture (14).

Various nature-inspired optimization algorithms have been applied to the problem of fECG extraction (see Section Theoretical Background). However, it is still unclear which type of optimization method is most suitable, as existing studies often use different experimental setups, making it difficult to compare results across studies. This limitation arises for several reasons. First, many published studies (15–17) evaluate only a single optimiser. Second, several studies (15–18) rely on synthetic signals or on datasets with limited complexity, which restricts the generalizability of their conclusions to real abdominal recordings. Third, because these methods are stochastic, their performance may vary across repeated runs, which is important to consider when comparing their behavior within the same extraction pipeline. This is particularly relevant in SA-based extraction, where the optimiser directly controls maternal template scaling and thereby influences the effectiveness of maternal ECG (mECG) suppression and the quality of subsequent fetal QRS detection.

To address this gap, this study performs a comparative evaluation of five population-based metaheuristics—artificial bee colony (ABC) (19), gray wolf optimization (GWO) (20), moth flame optimization (MFO) (21), particle swarm optimization (PSO) (22), and whale optimization algorithm (WOA) (23)—within the same sequential analysis framework for NI-fECG extraction. These algorithms were selected not only because they are widely used, but also because they are well established in the optimization literature, represent different search strategies, and can be applied in their original form. The selected methods cover a range of search behaviors, including probabilistic solution construction (ABC), swarm-based movement (PSO), and leader-based or spiral search strategies (GWO, WOA, and MFO). This diversity allows for a comparison of different approaches to balancing exploration and exploitation within the same framework. Because all methods are applied in their original form within a common extraction framework, a fair comparison under identical conditions is ensured. While this set of algorithms is not exhaustive, it provides a representative baseline for evaluating how the choice of optimization method affects fECG extraction from abdominal electrocardiogram (aECG) signals, which contain both fetal (fECG) and maternal (mECG) components.

Within this common optimization framework, the present study evaluates NI-fECG extraction performance under identical processing conditions using a unified and controlled assessment protocol. Performance is quantified by comparing automatically detected fetal QRS complexes with expert-annotated reference positions from real clinical datasets. Although CTG remains the clinical gold standard for routine fetal monitoring, it is typically evaluated according to its ability to detect fetal hypoxia or acidosis rather than according to signal extraction accuracy itself. In contrast, this study focuses specifically on the accuracy of NI-fECG extraction. While no universally accepted threshold for clinically sufficient NI-fECG extraction performance has been established, previous studies (24) generally regard fetal QRS detection rates above approximately 90% as highly effective. The objective of this study is therefore to provide an objective comparison of representative population-based optimization methods for NI-fECG extraction within the same sequential analysis framework and under identical experimental conditions.

## 2 Theoretical background

This section delves into the previously conducted studies in the field of fECG extraction using nature-based algorithms. Table 1 provides the reader with a quick overview of the written content.

**Table 1:** Summary of state-of-the-art literature in nature-based algorithms in the field of fECG extraction.

References	Algorithm	Data	Evaluation parameters
Ali et al. (15)	GA with adaptive filter	SISTA/DAISY	No statistical evaluation performed
Sargolzaei et al. (16)	PSO with ANFIS	SISTA/DAISY	Percent root-mean square
Jibia et al. (18)	MFO and fruit fly optimiser with adaptive filter	SISTA/DAISY	Mean square error and signal-to-noise ratio
Jibia et al. (17)	MFO with adaptive filter	Synthetic data	Mean square error and signal-to-noise ratio
Raj et al. (25)	GWO with SA	Labor and pregnancy	Evaluation based on ACC, SE, PPV, and F1.

Summary of state-of-the-art literature in nature-based algorithms in the field of fECG extraction.

In recent years, the combination of different adaptive techniques and training algorithms through integration or hybridization to overcome several limitations and achieve better results has led to a plethora of new intelligent systems. Computational intelligence enables the development of intelligent hybrid methods using neural networks, fuzzy systems, and evolutionary computation. These methods have been applied in the field of fECG extraction, and various studies have explored different techniques to achieve an optimal solution to the problem (16, 26–29). This section delves into previously conducted studies in the field using computational intelligence.

In a study by Ali et al. (15), the authors proposed an fECG extraction method based on the genetic algorithm (GA) combined with an adaptive filter. GA is a powerful and stochastic search technique. The authors tested the efficacy of the algorithm using the SISTA/DaISY dataset, which consists of eight signals from one pregnant woman. These signals are made up of three thoracic and five abdominal signals (30). However, the dataset used (SISTA/DaISY) has limitations, including a single recording with a low sampling frequency and insufficient segment length, making the fECG extraction relatively simple (14).

In another study by Sargolzaei et al. (16), PSO was used in conjunction with an adaptive neuro-fuzzy inference system (ANFIS) for fECG extraction. ANFIS is a complex algorithm that combines a fuzzy inference system, like Takagi-Sugeno, with a feed-forward neural network learning algorithm. The authors optimized the parameters required for ANFIS training using the PSO algorithm. They applied this method to both synthetic and real data from the SISTA/DaISY dataset.

Kockanat et al. (29) used differential evolution (DE) to optimize the weight coefficient vector in the LMS algorithm for fECG signal extraction. The authors also utilized clinical data from the SISTA/DaISY dataset.

Population-based algorithms were also employed in fECG signal extraction. Jibia et al. (17) used the Moth Flame Algorithm with LMS for fECG extraction, also using the SISTA/DaISY dataset. However, it should be noted that this dataset's limitations might impact the algorithm's effectiveness.

In another study by Jibia et al. (18), a comparison between two nature-based algorithms, the Moth Flame Optimization Algorithm, and the Fruit Fly Optimization Algorithm, was performed with an adaptive filter. The authors generated an ECG signal to demonstrate the efficacy of the algorithms. However, using synthetic signals may not fully represent the disturbances present in real ECG signals, limiting the conclusion's validity.

Raj et al. (25) utilized the gray wolf optimiser (GWO) with sequential analysis (SA) to find the optimum value of the scaling vector for creating an adaptive template of the maternal mECG. This template was used to eliminate the maternal template from the aECG and obtain the final fECG signal. The authors used two different databases consisting of real signals, available in the figshare repository (31). However, it should be noted that this algorithm's drawback is high computational time.

In summary, previous studies indicate that nature-inspired optimization can support NI-fECG extraction; however, the available evidence is difficult to compare across publications because the investigated systems differ in pipeline design and often rely on synthetic or otherwise limited datasets. As a result, it remains difficult to determine to what extent the observed performance differences are related to the optimization method itself rather than to the surrounding extraction framework or evaluation conditions. This motivates the present study, which compares representative population-based optimization methods within the same SA-based NI-fECG extraction framework.

## 3 Materials and methods

This section presents a comprehensive integration of population-based algorithms with the sequential analysis (SA) method. Population-based algorithms are first introduced, followed by an in-depth description of the SA method. Furthermore, details concerning the datasets utilized in this study are provided, and an outline of the evaluation protocol adopted for conducting the comparative analysis is presented. All the methods were performed in adherence to the relevant guidelines and regulatory standards.

### 3.1 Population-based algorithms

The optimization methods included in this study were selected as representative population-based approaches based on three criteria: (i) established use in the optimization literature, (ii) diversity of search mechanisms, and (iii) availability in their original unmodified formulations. This selection enables a consistent comparison within the same SA-based NI-fECG extraction framework without the confounding effects of hybridization or task-specific modifications. The selected methods are used in their original forms to preserve methodological comparability.

*Artificial bee colony*: Introduced in 2005 by Karaboga, this algorithm mimics the behavior of bees foraging for food or nectar. The exploration and exploitation of the food or a global solution is a mathematical model of the bees looking for the best nectar. Further information and mathematical model can be found in Karaboga (19).

*Gray wolf optimization*: Seyedali Mirjalili introduced the algorithm in 2014, and the optimization algorithm draws from the social hierarchy and the hunting mechanism from the operation methods of a pack of wolves. The algorithm follows the hunting technique of a pack of wolves given by Muro et al. (32) to achieve the best solution to the problem. The mathematical model of these techniques along with the exploration and exploitation phase of these search agents can be studied in Mirjalili et al. (20).

*Moth flame optimization*: This method was developed by Mirjalili (21). The algorithm is inspired by the navigation of moths at night using lunar cues from the moon, stars, or constellations. The navigation model to their destination is used to model the algorithm to find an optimal solution for the problem. Mirjalili (21) describes the algorithm at length along with the mathematical model of the search for an optimal solution.

*Particle swarm algorithm*: PSO is one of the most popular algorithms in the field of meta-heuristic algorithms. The algorithm is formulated by mimicking the behavior of social animals like swarms, fish or flocks of birds. Further information on the algorithm, settings and parameters can be found in Kennedy and Eberhart (22).

*Whale optimization algorithm*: Proposed in 2016, the algorithm depicts the hunting behavior of the humpback whale. The hunting mechanism is called bubble net feeding. This hunting technique is unique to humpback whales. The algorithm created by Mirjalili and Lewis gives the model that uses the technique found in nature to obtain an optimal solution to the problem in the discussion. The detailed background of the algorithm is given in Mirjalili and Lewis (23).

### 3.2 Sequential analysis

The SA method is well-known in the field of fECG extraction and has been proven to be robust in delivering well-extracted signals. In this paper, we optimize the scaling vector with the use of various population-based algorithms. The objective is to create a template that resembles the mECG signal and then eliminate it from aECG, which consists of both the mECG and fECG signals.

The SA method was introduced by Martens et al. (33) and uses a *priori* information about the maternal peaks. This method uses this information to detect the maternal peaks and creates a template of the mECG. As mentioned earlier, the mECG signal experiences morphological changes and hence, the created template requires a scaling procedure to adapt well. This is helpful to have an efficient elimination of the mECG. The mECG is made up of three isolated parts: the P-wave, the QRS complex, and the T-wave, and each part has to be scaled in accordance with the original signal. The total length of the mECG window is 0.70 s. The mECG window is divided into the following section:

- μ_*R*_ – samples between 0.05 s before and after an R peak detected is considered QRS complexes.
- μ_*P*_ – samples before 0.20 s before the QRS complex are considered P-waves.
- μ_*T*_ – samples before 0.40 s after the QRS complex are considered T-waves.

The matrix with the P-wave, QRS complex and T-wave vectors is defined as:


$$\begin{array}{l} M=\left(\begin{array}{ccc} | & 0 & 0\\ \mu_{p} & 0 & 0\\ | & 0 & 0\\ 0 & | & 0\\ 0 & \mu_{QRS} & 0\\ 0 & | & 0\\ 0 & 0 & |\\ 0 & 0 & \mu_{T}\\ 0 & 0 & | \end{array}\right). \end{array}$$ (1)

The mECG complex, $\hat{m}$ is represented as $\hat{m}=Ma$, where *a* is a scaling vector, *a*=(*a*_*P*_ *a*_*QRS*_ *a*_*T*_). The value of *a* for each vector is given by:


$$\begin{array}{l} a={\left(M^{T}M\right)}^{-1}M^{T}m. \end{array}$$ (2)

The objective is to minimize the error, which is given by:


$$\begin{array}{l} e^{2}=min|\mu a-m{|}^{2}. \end{array}$$ (3)

Once the maternal template is created, it is used to eliminate the mECG from aECG. Following this, a QRS detector is used to detect fetal peaks.

As mentioned above, the scaling factor is crucial in creating an mECG. The authors propose using the various swarm optimization methods to optimize the scaling factor and determine the efficacy of the algorithms.

### 3.3 Datasets

The datasets, denoted as *Pregnancy* and *Labor*, utilized in this study comprise *real* signals and are publicly available on a server. These datasets were recorded as part of research projects conducted at the Department of Obstetrics and Gynecology, Medical University of Silesia in Katowice, Poland. The research received approval from the University's Bioethics Committee (Commission approval number NN-013-345/02), and all participants provided written consent to participate in the study. Detailed information about the datasets can be found in Matonia et al. (31).

For both datasets, the aECG signals were obtained from the maternal abdomen using the KOMPOREL system. The sensing electrodes were positioned around the navel line, with a common electrode placed over the pubic symphysis and a reference electrode on the maternal left leg. The direct fECG signals in Labor dataset were recorded invasively from the fetal head using a sterile spiral electrode. The signals were digitized with a 16-bit resolution and sampled at a frequency of 500 Hz for aECG signals and 1,000 Hz for direct fECG signals.

The Labor dataset comprises 12 antepartum records, each lasting for 5 min, obtained from women in their 38^*th*^ to 42^*nd*^ week of pregnancy. Each record includes 4 aECG signals and a simultaneous direct fECG signal recorded from the fetal scalp using a scalp electrode. In contrast, the Pregnancy dataset consists of 10 records, each lasting for 20 min, obtained from women in their 32^*nd*^ to 42^*nd*^ week of pregnancy. Each record in this dataset includes 4 aECG signals but does not include any direct fECG signal. Both datasets provide annotations of the exact fQRS complex position, which were determined through R-peak detection. The accuracy of the annotations was confirmed by clinical experts.

The selected datasets consist of real aECG recordings with expert annotations of fetal QRS complexes, which enables an objective evaluation of extraction accuracy. The availability of such annotated real-world datasets is limited, and their use allows a consistent quantitative comparison of the evaluated methods. It should also be noted that the utilized datasets are relatively limited in size and originate from a single acquisition system and clinical setting, which may affect the generalizability of the obtained results.

### 3.4 Evaluation protocol

The performance of the proposed algorithms was evaluated by determining the accuracy of R-peak detection, which is one of the most common approaches used in the field of fECG signal processing (34, 35). The accuracy of the detection was calculated as follows:


$$\begin{array}{l} ACC=\frac{TP}{TP+FP+FN}\cdot 100\text{(\%)}, \end{array}$$ (4)

where TP (true positive) indicates the correctly detected fetal R-peak (detected ±50 ms before or after the corresponding reference fetal R-peak in the annotation), FP (false positive) represents incorrect detection of the presence of fetal R-peak, and FN (false negative) denotes undetected fetal R-peak.

Theoretically, given the high fHR, a lower limit as the aforementioned 50 ms could have been chosen. However, in this study, we sought to adhere to an evaluation method similar to that used in other literature focusing on fetal ECG extraction and subsequent R-peak detection (36). Adherence to this standardized evaluation criterion ensures direct comparability of the results obtained with previously published study results. Furthermore, preliminary observations indicated that the actual accuracy of R-peak detection using the CWT detector in combination with the proposed fECG extraction approaches is very high, and the error relative to the reference annotations typically falls within a range of a few samples. This confirms that the ± 50 ms limit serves for comparability between studies, not as compensation for inaccurate detections.

Accuracy was chosen as the primary metric for testing, but we also calculated sensitivity (SE), positive predictive value (PPV), and the F1, which represents the harmonic mean between SE and PPV:


$$\begin{array}{l} SE=\frac{TP}{TP+FN}\cdot 100\text{(\%)}. \end{array}$$ (5)

$$\begin{array}{l} PPV=\frac{TP}{TP+FP}\cdot 100\text{(\%)}. \end{array}$$ (6)

$$\begin{array}{l} F1=2\cdot \frac{SE\cdot PPV}{SE+PPV}\text{(\%)}. \end{array}$$ (7)

Because the algorithms were stochastic and each experiment was repeated 30 times independently, the distributions of ACC values were summarized using medians and 95% confidence intervals (CI). Some CI could not be calculated due to insufficient variability in the data. This choice was motivated by the presence of non-normal and asymmetric ACC distributions observed in some experimental conditions. To avoid interpreting statistical significance without information about practical relevance, effect sizes were reported together with p-values. For the Friedman test, Kendall's W was used as an effect size measure. For pairwise comparisons, paired Cohen's d was calculated using record-level median ACC values.

## 4 Experimental setup

**Figure 1:**
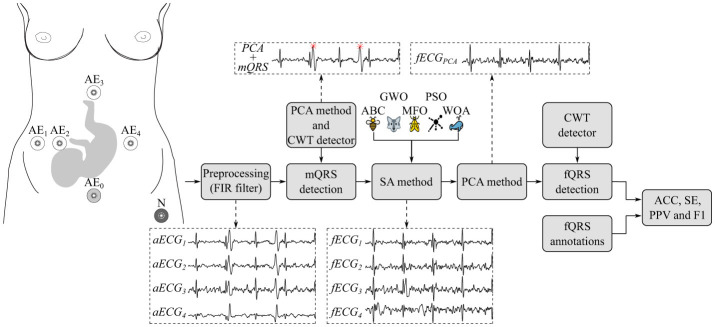
Block diagram of the proposed experiment (the example signals are used from recording 8 of the Labor dataset).

To ensure a consistent comparison, all evaluated optimization methods were integrated into the same extraction pipeline and assessed under identical processing and evaluation conditions. The block scheme depicted in Figure 1 outlines the proposed approach of integrating SA with the aforementioned population-based algorithms for fECG extraction. This integration aims to enhance the overall effectiveness of the extraction process. Below is the description of each block.

The datasets used are namely Labor and Pregnancy dataset, which are mentioned in the previous section. The experiment focuses on preparing aECG and mECG signals for the application of the optimization algorithm to estimate the fECG signal. Figure 1 illustrates the step-by-step procedure, commencing with the utilization of signals measured by abdominal electrodes (AE_1_–AE_4_) as input.

After the measurement of the signal, in the next step, the input signals (abdominal ECGs) first go through pre-processing, which removes baseline wander and power-line interference from the input signals.

The next stage is to detect the maternal QRS peaks to create templates that would match the individual parts of the mECG cycle: P wave, QRS complex, and T wave, leading to their elimination in the next stage. The PCA method is used to select the principle component from the abdominal signals corresponding to mECG.

The following stage is the integration of SA with the optimization algorithms, which would provide the optimal matrix values to create a template that matches the time-varying morphology of the mECG signal.

The integration of the swarm-based optimiser with SA follows these steps:

**Step 1:** Initialization of search agents/particles/bees with random position values for the scaling vector, *a*.

**Step 2:** The optimiser runs independently for each detected mECG to optimize the scaling factors for different parts of the mECG cycle, including the P wave, QRS complex, and T wave. These scaling factors represent the positions of the lead agent, best particle, or bee.

**Step 3:** The optimization process yields an mECG template, which is then employed to remove the maternal component from the input signal.

**Step 4:** The objective function utilized by the optimiser is as follows:


$$\begin{array}{l} J=\min\left(\overset{N}{\underset{n=1}{\sum }}{\left(input\;signal-mECG\;template\right)}^{2}\right). \end{array}$$ (8)

$$\begin{array}{l} J=MSE\left(input\;signal-mECG\;template\right). \end{array}$$ (9)

The overall algorithmic flow of the proposed fECG extraction framework is summarized in pseudo-code form in Algorithm 1. The proper configuration of input parameters is critical for the performance of tested population-based algorithms. Based on recommendations from the original literature (19–23), preliminary empirical testing and the direct expert knowledge of the co-authors, the algorithm-specific parameters were set as detailed in Table 2. Furthermore, to evaluate the scalability and stability of the extraction systems under different computational budgets, two distinct experimental configurations were designed for the population size and maximum iterations. The first configuration set both parameters to 10, while the second configuration set both to 20. Given the stochastic nature of these methods, each experimental setup was executed 30 times independently.

Algorithm 1 General algorithmic flow: fECG extraction via SA and population-based algorithms. 
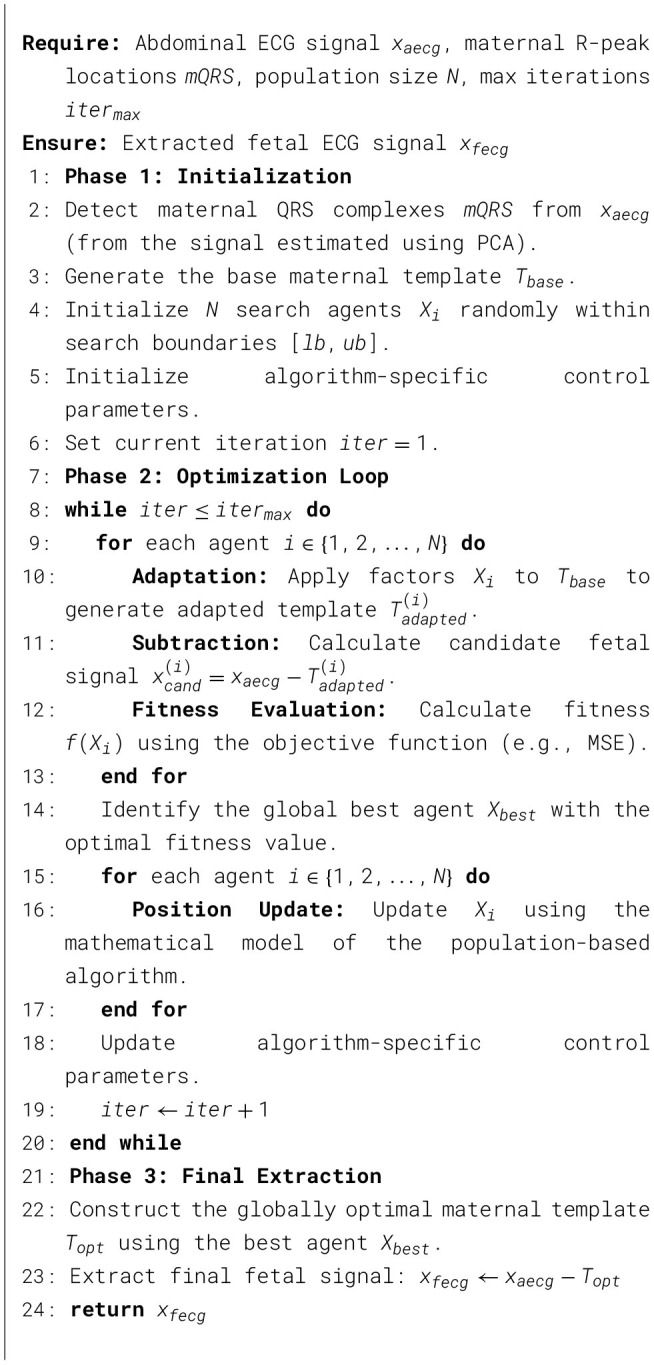

**Table 2:** Selected and tested parameters for individual population-based algorithms.

Algorithm	Parameter	Value	Notes
All methods	Population size (*N*)	10 or 20	Two configurations were tested.
	Maximum iterations (*iter*)	10 or 20	Two configurations were tested.
	Dimensions (*d*)	3	Scaling factors for P-wave, QRS-complex, and T-wave.
	Search space boundaries	[0,3]	Constraints for scaling coefficients.
ABC	Trial limit (*L*)	18 or 36	Calculated as: 0.6·*d*·*N*.
GWO	Convergence parameter (*a*)	2 → 0	Linearly decreased over iterations.
MFO	Convergence parameter (*a*)	−1 → −2	Linearly decreased over iterations.
	Spiral constant (*b*)	1	Defines the shape of the logarithmic spiral.
	Number of flames	*N* → 1	Linearly decreased from initial *N* to 1.
PSO	Inertia weight (*w*)	0.9 → 0.2	Linearly decreased over iterations.
	Cognitive constant (*c*_1_)	2	Personal learning coefficient.
	Social constant (*c*_2_)	2	Global learning coefficient.
	Max velocity (*v*_*max*_)	0.6	Limited to 20% of the search area.
WOA	Convergence parameter (*a*)	2 → 0	Linearly decreased over iterations.
	Spiral parameter (*a*_*spiral*_)	−1 → −2	Linearly decreased to calculate spiral position.
	Spiral constant (*b*)	1	Defines the shape of the logarithmic spiral.

Selected and tested parameters for individual population-based algorithms.

The PCA method is used again to enhance the fECG component in the estimated signal to further improve the fHR detection. Following fECG estimation, R-peaks are detected using the continuous wavelet transform (CWT) detector, and a statistical analysis of R-peaks accuracy is conducted with respect to the reference annotations. The entire experiment is repeated 30 times for each method to demonstrate their effectiveness and stability.

This detector performs signal decomposition up to the fifth level using the gaus1 wavelet. After applying the CWT, all local minima and maxima in the signal are identified and adaptively refined. In the following step, zero crossings are located between each pair of adjacent local minima and maxima where the time interval is less than 120 ms. Finally, the most prominent peak to the right of each identified zero crossing is located and marked as the R-peak.

To ensure accurate reproducibility, the random number generators in each population-based algorithms were initialized with fixed seed values before each run. All algorithms and the sequential analysis (SA) framework were implemented in MATLAB R2023a and run on a workstation equipped with Intel i9-10980XE, NVIDIA GeForce GTX 1050 Ti and 64 GB of RAM.

## 5 Results

The results are presented with the aim of comparing the extraction accuracy achieved by individual optimization methods under identical experimental conditions within the same SA-based NI-fECG extraction framework. The algorithms were run independently 30 times for each recording in the two datasets (Labor and Pregnancy). Algorithm performance evaluation was performed by determining the accuracy of R-peak detection using ACC parameter. Table 3 shows the median of ACC values along with the first quartile (Q1) and third quartile (Q3) values for all tested algorithms.

**Table 3:** Statistical summary of ACC (%) for the Labor and Pregnancy datasets, presented as median (95% CI) over 30 runs.

Dataset	Rec.	ABC	GWO	MFO	PSO	WOA
**Labor**	r01	59.29 (9.55; 64.90)	99.23 (—; —)	99.23 (98.15; 99.23)	99.23 (—; —)	99.23 (—; —)
	r02	58.41 (35.61; 60.34)	95.04 (—; —)	95.04 (94.38; 95.04)	95.04 (95.04; 95.04)	95.04 (—; —)
	r03	27.58 (15.69; 27.98)	52.75 (52.24; 53.35)	52.91 (52.37; 53.86)	53.52 (53.19; 53.70)	52.14 (51.66; 52.31)
	r04	30.88 (12.93; 57.92)	95.15 (94.87; 95.43)	95.29 (94.74; 95.64)	95.29 (95.01; 95.50)	95.15 (94.81; 95.29)
	r05	71.84 (66.60; 73.78)	97.60 (—; —)	97.60 (97.60; 97.60)	97.60 (—; —)	97.75 (97.60; 97.90)
	r06	72.56 (16.27; 75.11)	97.83 (97.83; 98.26)	98.12 (97.98; 98.26)	98.05 (97.91; 98.26)	98.26 (98.26; 98.41)
	r07	51.80 (50.00; 54.25)	90.65 (90.35; 91.07)	91.23 (91.00; 91.66)	90.92 (90.63; 90.92)	90.21 (89.98; 90.42)
	r08	75.80 (67.14; 78.10)	99.84 (—; —)	99.84 (99.84; 99.84)	99.84 (—; —)	99.84 (—; —)
	r09	66.25 (33.13; 68.61)	96.07 (95.78; 96.36)	95.78 (95.43; 96.07)	95.78 (95.78; 96.07)	95.93 (95.78; 96.07)
	r10	21.65 (8.63; 57.73)	99.36 (99.05; 99.36)	99.05 (99.05; 99.20)	99.05 (99.05; 99.36)	99.05 (99.05; 99.36)
	r11	58.81 (55.01; 61.36)	96.97 (96.67; 96.97)	96.97 (96.97; 96.97)	96.97 (96.67; 96.97)	96.97 (96.82; 97.26)
	r12	85.60 (84.44; 86.33)	98.94 (—; —)	98.64 (98.64; 98.94)	98.94 (—; —)	98.94 (—; —)
**Pregnancy**	r01	14.35 (12.09; 30.01)	98.73 (98.72; 98.75)	98.76 (98.73; 98.82)	98.79 (98.73; 98.79)	98.35 (98.31; 98.51)
	r02	45.06 (19.84; 47.37)	97.75 (97.68; 97.82)	97.82 (97.78; 97.88)	97.85 (97.85; 97.99)	97.68 (97.54; 97.78)
	r03	28.62 (18.45; 30.16)	94.58 (94.56; 94.64)	94.71 (94.56; 94.82)	94.67 (94.56; 94.75)	94.86 (94.77; 95.06)
	r04	14.98 (6.51; 54.98)	99.28 (—; —)	99.28 (99.28; 99.28)	99.28 (—; —)	99.28 (99.28; 99.28)
	r05	14.03 (8.77; 34.34)	99.17 (99.10; 99.28)	99.28 (99.21; 99.28)	99.35 (99.35; 99.35)	99.07 (98.96; 99.16)
	r06	37.82 (13.40; 42.67)	91.42 (91.35; 91.50)	91.56 (91.48; 91.60)	91.51 (91.50; 91.57)	91.07 (90.68; 91.32)
	r07	33.52 (10.42; 38.31)	91.66 (91.56; 91.72)	91.72 (91.59; 91.78)	91.69 (91.66; 91.78)	91.45 (91.31; 91.59)
	r08	25.87 (11.02; 35.89)	95.00 (94.91; 95.10)	94.97 (94.89; 95.13)	95.08 (95.08; 95.08)	94.47 (94.25; 94.64)
	r09	12.38 (11.55; 12.96)	96.83 (96.71; 96.88)	96.75 (96.68; 96.85)	96.51 (96.44; 96.61)	96.95 (96.84; 97.05)
	r10	20.52 (11.48; 21.97)	88.00 (87.84; 88.08)	88.00 (87.91; 88.12)	88.02 (87.64; 88.10)	88.44 (88.27; 88.56)

Statistical summary of ACC (%) for the Labor and Pregnancy datasets, presented as median (95% CI) over 30 runs.

The performance of the GWO, MFO, PSO, and WOA algorithms was comparable for all records from both datasets, as the values of the median of ACC did not differ by more than 1.5%. For both datasets, all four methods were highly effective (median of ACC > 90%) for all records except record r10 from the Pregnancy dataset, where the value of the median of ACC was slightly lower (in the range 88.00%–88.44%) and record r03 from the Labor dataset, for which all methods achieved a median of ACC < 54%. The ABC algorithm was significantly different from the performance of these four algorithms, whose median of ACC values were significantly lower for all records from both datasets (in some cases by more than 80%). At the same time, the ABC algorithm can be considered the least effective, as it was able to effectively extract fECG only for record r12 from the Labor dataset, with the median of ACC = 85.60%. For the other records, these values were significantly lower and the algorithm cannot be considered successful for them.

Furthermore, an evaluation was also performed based on SE (Table 4), PPV (Table 5), and F1 (Table 6). Based on these parameters, it can be seen that on both datasets, the GWO, MFO, PSO, and WOA methods achieved high performance (median of SE, PPV, and F1 > 90%) on all records except record r03 from the Labor dataset (SE < 75%, PPV < 66%, and F1 < 70%). This record can therefore be identified as the lowest quality for fECG processing and extraction across both datasets. As for the ABC method, it again achieved the worst results in terms of SE, PPV, and F1. On the Pregnancy dataset, this method did not achieve an SE, PPV, or F1 > 80% for any record. On the Labor dataset, this method achieved an SE > 80% on 5 records, a PPV > 80% on 4 records, and an F1 > 80% on 4 records.

**Table 4:** Statistical summary of SE (%) for the Labor and Pregnancy datasets, presented as median (95% CI) over 30 runs.

Dataset	Rec.	ABC	GWO	MFO	PSO	WOA
**Labor**	r01	75.93 (12.27; 79.42)	99.69 (—; —)	99.69 (99.07; 99.69)	99.69 (—; —)	99.69 (—; —)
	r02	75.75 (50.16; 77.86)	99.22 (—; —)	99.22 (98.83; 99.22)	99.22 (99.22; 99.22)	99.22 (—; —)
	r03	48.05 (24.09; 50.21)	73.60 (73.25; 74.02)	73.81 (73.32; 74.58)	74.30 (74.02; 74.58)	73.32 (72.70; 73.46)
	r04	45.52 (18.50; 74.16)	97.94 (97.80; 98.09)	97.94 (97.80; 98.16)	97.94 (97.94; 98.09)	97.94 (97.80; 98.02)
	r05	83.48 (80.61; 84.62)	98.79 (—; —)	98.79 (98.79; 98.79)	98.79 (—; —)	98.87 (98.79; 98.94)
	r06	84.87 (22.73; 86.55)	98.98 (98.98; 99.27)	99.12 (99.12; 99.27)	99.12 (99.05; 99.27)	99.27 (99.19; 99.27)
	r07	69.38 (67.72; 71.68)	95.02 (94.86; 95.25)	95.41 (95.25; 95.57)	95.09 (94.94; 95.09)	94.78 (94.54; 94.86)
	r08	86.75 (81.16; 88.52)	99.84 (—; —)	99.84 (99.84; 99.84)	99.84 (—; —)	99.84 (—; —)
	r09	80.49 (44.36; 82.05)	97.92 (97.77; 98.07)	97.77 (97.63; 97.92)	97.77 (97.77; 97.92)	97.92 (97.77; 97.92)
	r10	32.70 (11.57; 74.72)	99.68 (99.52; 99.68)	99.52 (99.52; 99.60)	99.52 (99.52; 99.68)	99.52 (99.52; 99.68)
	r11	76.09 (73.07; 78.02)	98.92 (98.76; 98.92)	98.92 (98.92; 98.92)	98.92 (98.76; 98.92)	98.92 (98.84; 99.07)
	r12	92.31 (91.71; 92.84)	99.54 (—; —)	99.39 (99.39; 99.54)	99.54 (—; —)	99.54 (—; —)
**Pregnancy**	r01	20.87 (17.53; 49.17)	99.42 (99.42; 99.42)	99.44 (99.42; 99.46)	99.46 (99.42; 99.46)	99.23 (99.20; 99.29)
	r02	63.00 (30.25; 64.44)	98.97 (98.93; 99.00)	99.00 (98.97; 99.02)	99.00 (99.00; 99.07)	98.93 (98.89; 99.00)
	r03	50.08 (33.80; 51.23)	96.98 (96.96; 97.00)	97.04 (96.96; 97.12)	97.04 (96.96; 97.08)	97.15 (97.08; 97.23)
	r04	20.68 (7.84; 72.19)	99.64 (—; —)	99.64 (99.64; 99.64)	99.64 (—; —)	99.64 (99.64; 99.64)
	r05	21.30 (11.30; 54.80)	99.64 (99.60; 99.68)	99.68 (99.64; 99.68)	99.71 (99.71; 99.71)	99.57 (99.53; 99.60)
	r06	57.63 (18.87; 62.16)	95.85 (95.81; 95.88)	95.90 (95.88; 95.92)	95.88 (95.88; 95.92)	95.67 (95.48; 95.80)
	r07	51.41 (13.79; 57.42)	96.00 (95.99; 96.05)	96.02 (95.97; 96.05)	95.99 (95.99; 96.05)	95.95 (95.84; 96.02)
	r08	40.43 (15.55; 55.38)	97.63 (97.58; 97.66)	97.63 (97.56; 97.66)	97.66 (97.66; 97.66)	97.33 (97.22; 97.46)
	r09	19.23 (18.21; 20.03)	98.57 (98.50; 98.57)	98.52 (98.50; 98.57)	98.43 (98.39; 98.46)	98.60 (98.53; 98.66)
	r10	37.17 (17.65; 41.80)	95.58 (95.49; 95.66)	95.58 (95.52; 95.66)	95.60 (95.39; 95.68)	95.87 (95.76; 95.91)

Statistical summary of SE (%) for the Labor and Pregnancy datasets, presented as median (95% CI) over 30 runs.

**Table 5:** Statistical summary of PPV (%) for the Labor and Pregnancy datasets, presented as median (95% CI) over 30 runs.

Dataset	Rec.	ABC	GWO	MFO	PSO	WOA
**Labor**	r01	72.69 (31.71; 77.85)	99.53 (—; —)	99.53 (99.07; 99.53)	99.53 (—; —)	99.53 (—; —)
	r02	71.69 (51.67; 73.25)	95.76 (—; —)	95.76 (95.45; 95.76)	95.76 (95.76; 95.76)	95.76 (—; —)
	r03	38.05 (32.00; 39.94)	65.10 (64.60; 65.50)	65.11 (64.66; 65.91)	65.64 (65.39; 65.80)	64.47 (64.05; 64.58)
	r04	48.41 (31.55; 72.55)	97.09 (96.94; 97.23)	97.16 (96.88; 97.38)	97.23 (97.02; 97.31)	97.09 (96.87; 97.09)
	r05	83.66 (79.41; 84.89)	98.79 (—; —)	98.79 (98.79; 98.79)	98.79 (—; —)	98.87 (98.79; 98.94)
	r06	83.34 (36.41; 84.90)	98.83 (98.83; 98.98)	98.98 (98.83; 98.98)	98.91 (98.83; 98.98)	98.98 (98.98; 99.12)
	r07	67.16 (64.98; 69.06)	95.17 (94.94; 95.55)	95.56 (95.33; 95.72)	95.40 (95.24; 95.40)	94.93 (94.91; 95.09)
	r08	85.57 (79.50; 86.90)	100.00 (—; —)	100.00 (100.00; 100.00)	100.00 (—; —)	100.00 (—; —)
	r09	78.72 (51.33; 80.73)	98.07 (97.92; 98.22)	97.92 (97.70; 98.07)	97.92 (97.92; 98.07)	97.92 (97.92; 98.07)
	r10	38.44 (27.18; 71.91)	99.68 (99.52; 99.68)	99.52 (99.52; 99.60)	99.52 (99.52; 99.68)	99.52 (99.52; 99.68)
	r11	72.55 (69.01; 74.48)	98.01 (97.85; 98.01)	98.01 (98.01; 98.01)	98.01 (97.85; 98.01)	98.01 (97.93; 98.16)
	r12	92.11 (91.50; 92.43)	99.39 (—; —)	99.24 (99.24; 99.39)	99.39 (—; —)	99.39 (—; —)
**Pregnancy**	r01	31.40 (28.55; 43.84)	99.30 (99.30; 99.31)	99.31 (99.30; 99.36)	99.33 (99.30; 99.33)	99.12 (99.07; 99.20)
	r02	61.28 (36.61; 63.72)	98.75 (98.72; 98.79)	98.79 (98.79; 98.84)	98.83 (98.83; 98.90)	98.72 (98.61; 98.75)
	r03	39.89 (31.39; 42.59)	97.45 (97.45; 97.49)	97.53 (97.45; 97.57)	97.49 (97.45; 97.53)	97.61 (97.53; 97.71)
	r04	36.81 (29.04; 69.84)	99.64 (—; —)	99.64 (99.64; 99.64)	99.64 (—; —)	99.64 (99.64; 99.64)
	r05	29.34 (27.90; 50.53)	99.53 (99.50; 99.60)	99.60 (99.60; 99.62)	99.64 (99.64; 99.64)	99.49 (99.42; 99.53)
	r06	53.10 (31.55; 58.45)	95.19 (95.15; 95.24)	95.26 (95.22; 95.31)	95.25 (95.23; 95.29)	94.97 (94.76; 95.12)
	r07	49.03 (32.16; 53.09)	95.31 (95.23; 95.35)	95.33 (95.28; 95.38)	95.34 (95.31; 95.38)	95.10 (95.06; 95.22)
	r08	41.74 (28.32; 50.22)	97.26 (97.21; 97.31)	97.22 (97.17; 97.34)	97.30 (97.30; 97.30)	96.96 (96.86; 97.07)
	r09	25.93 (25.21; 26.61)	98.21 (98.16; 98.26)	98.17 (98.12; 98.23)	98.02 (97.98; 98.09)	98.31 (98.25; 98.37)
	r10	29.20 (24.73; 32.77)	91.71 (91.65; 91.75)	91.74 (91.70; 91.81)	91.72 (91.52; 91.75)	91.92 (91.85; 92.01)

Statistical summary of PPV (%) for the Labor and Pregnancy datasets, presented as median (95% CI) over 30 runs.

**Table 6:** Statistical summary of F1 (%) for the Labor and Pregnancy datasets, presented as median (95% CI) over 30 runs.

Dataset	Rec.	ABC	GWO	MFO	PSO	WOA
**Labor**	r01	74.43 (17.44; 78.70)	99.61 (—; —)	99.61 (99.07; 99.61)	99.61 (—; —)	99.61 (—; —)
	r02	73.75 (50.79; 75.26)	97.46 (—; —)	97.46 (97.11; 97.46)	97.46 (97.46; 97.46)	97.46 (—; —)
	r03	43.23 (27.11; 43.73)	69.06 (68.63; 69.58)	69.20 (68.75; 70.01)	69.73 (69.44; 69.87)	68.54 (68.12; 68.69)
	r04	46.90 (22.89; 73.34)	97.51 (97.37; 97.66)	97.58 (97.30; 97.77)	97.58 (97.44; 97.69)	97.51 (97.34; 97.59)
	r05	83.61 (79.94; 84.91)	98.79 (—; —)	98.79 (98.79; 98.79)	98.79 (—; —)	98.87 (98.79; 98.94)
	r06	84.10 (27.98; 85.79)	98.90 (98.90; 99.12)	99.05 (98.97; 99.12)	99.01 (98.94; 99.12)	99.12 (99.12; 99.19)
	r07	68.25 (66.67; 70.34)	95.09 (94.93; 95.32)	95.41 (95.28; 95.65)	95.24 (95.09; 95.24)	94.85 (94.72; 94.97)
	r08	86.23 (80.32; 87.70)	99.92 (—; —)	99.92 (99.92; 99.92)	99.92 (—; —)	99.92 (—; —)
	r09	79.69 (46.02; 81.38)	97.99 (97.84; 98.14)	97.84 (97.66; 97.99)	97.84 (97.84; 97.99)	97.92 (97.84; 97.99)
	r10	35.16 (15.87; 73.20)	99.68 (99.52; 99.68)	99.52 (99.52; 99.60)	99.52 (99.52; 99.68)	99.52 (99.52; 99.68)
	r11	74.07 (70.98; 76.05)	98.46 (98.30; 98.46)	98.46 (98.46; 98.46)	98.46 (98.30; 98.46)	98.46 (98.38; 98.61)
	r12	92.25 (91.56; 92.66)	99.46 (—; —)	99.31 (99.31; 99.46)	99.46 (—; —)	99.46 (—; —)
**Pregnancy**	r01	25.05 (21.57; 46.16)	99.36 (99.35; 99.37)	99.38 (99.36; 99.41)	99.39 (99.36; 99.39)	99.16 (99.14; 99.24)
	r02	62.13 (33.12; 64.28)	98.86 (98.82; 98.89)	98.89 (98.88; 98.93)	98.91 (98.91; 98.98)	98.82 (98.75; 98.87)
	r03	44.50 (30.88; 46.34)	97.21 (97.20; 97.24)	97.28 (97.20; 97.34)	97.26 (97.20; 97.30)	97.36 (97.31; 97.47)
	r04	25.89 (12.22; 70.95)	99.64 (—; —)	99.64 (99.64; 99.64)	99.64 (—; —)	99.64 (99.64; 99.64)
	r05	24.60 (16.12; 51.12)	99.58 (99.55; 99.64)	99.64 (99.60; 99.64)	99.67 (99.67; 99.67)	99.53 (99.47; 99.57)
	r06	54.88 (23.63; 59.82)	95.52 (95.48; 95.56)	95.59 (95.55; 95.62)	95.56 (95.56; 95.60)	95.33 (95.11; 95.46)
	r07	50.16 (18.82; 55.40)	95.65 (95.60; 95.68)	95.67 (95.60; 95.71)	95.66 (95.65; 95.71)	95.53 (95.45; 95.61)
	r08	41.07 (19.84; 52.83)	97.44 (97.38; 97.49)	97.42 (97.37; 97.50)	97.48 (97.48; 97.48)	97.15 (97.04; 97.24)
	r09	22.04 (20.71; 22.96)	98.39 (98.33; 98.41)	98.34 (98.31; 98.40)	98.22 (98.18; 98.27)	98.45 (98.39; 98.50)
	r10	34.05 (20.59; 36.02)	93.61 (93.53; 93.66)	93.61 (93.56; 93.69)	93.62 (93.41; 93.67)	93.86 (93.77; 93.94)

Statistical summary of F1 (%) for the Labor and Pregnancy datasets, presented as median (95% CI) over 30 runs.

To statistically compare the accuracy of extraction (ACC) of algorithms, the variability of ACC depending on the algorithm used was first compared for both databases and for all records. Based on the Levene test (*p* < 0.001), it can be concluded that for all cases (all records in both databases) there is a statistically significant difference in the variability of ACC between the algorithms at the 0.05 significance level. Subsequently, the pairwise comparison (TukeyHSD test with correction for multiple comparisons applied to the residuals) showed that for all cases the variability in accuracy of the ABC algorithm is statistically significantly different from the variability of all other algorithms (GWO, MFO, PSO, and WOA) between which there is no statistically significant difference in this respect.

To quantify the practical importance of the observed differences in variability between algorithms, eta-squared (η^2^) effect sizes were calculated based on the analysis of absolute deviations from group medians. The obtained η^2^ values ranged approximately from 0.33 to 0.84 for the Labor dataset and from 0.45 to 0.66 for the Pregnancy dataset, indicating large to very large effects according to conventional interpretation thresholds. These findings demonstrate that a substantial proportion of the variability in ACC was explained by the choice of the optimization algorithm. In practical terms, the observed differences in stability between algorithms were not only statistically significant but also highly relevant. The large effect sizes were primarily driven by the markedly unstable behavior of the ABC algorithm, whereas GWO, MFO, PSO, and WOA exhibited consistently low variability across repeated runs.

The coefficient of variation of the accuracy of the ABC algorithm for 20 of the 22 records was in the range 41.7%–89.4%, while for the other algorithms the coefficient of variation of the accuracy for all records was no more than 2.5% (see Table 7). There are multiple other reasons for this behavior, including limitations of ABC, such as premature and slow convergence rate or falling into local optima (37–39). Other factors will be discussed in detail in section Discussion.

**Table 7:** Variation coefficient (%) of ACC for Labor and Pregnancy dataset.

Dataset	Rec.	ABC	GWO	MFO	PSO	WOA
**Labor**	r01	68.5	0.0	0.5	0.0	0.0
	r02	58.3	0.3	0.8	0.3	0.3
	r03	49.2	1.9	2.5	1.5	1.8
	r04	72.4	0.4	0.7	0.5	1.6
	r05	9.0	0.1	0.3	0.1	0.2
	r06	66.1	0.2	0.2	0.2	0.1
	r07	50.9	0.9	0.9	0.7	1.3
	r08	46.6	0.0	0.1	0.0	0.0
	r09	60.5	0.4	0.6	0.4	0.3
	r10	80.9	0.2	0.2	0.2	0.4
	r11	41.7	0.2	0.2	0.2	0.3
	r12	2.7	0.0	0.2	0.0	0.0
**Pregnancy**	r01	54.2	0.1	0.1	0.0	0.2
	r02	55.6	0.2	0.1	0.1	0.2
	r03	53.1	0.2	0.3	0.2	0.3
	r04	89.4	0.0	0.1	0.0	0.1
	r05	69.4	0.1	0.1	0.0	0.2
	r06	63.0	0.2	0.2	0.1	0.6
	r07	68.5	0.3	0.3	0.2	0.4
	r08	57.1	0.2	0.3	0.1	0.5
	r09	44.3	0.2	0.2	0.1	0.4
	r10	61.4	0.4	0.3	0.3	0.4

Regarding the SE, PPV, and F1 parameters, the results were similar to those for ACC (see the Tables 8, 9, 10). It is evident that, once again, the values of these parameters for the ABC method showed high variability. For 20 out of the 22 records (the same as those mentioned above for ACC), SE ranged from 38.8% to 88.5%, PPV from 10.1% to 49.8%, and F1 from 35.8% to 78.9%.

**Table 8:** Variation coefficient (%) of SE for Labor and Pregnancy dataset.

Dataset	Rec.	ABC	GWO	MFO	PSO	WOA
**Labor**	r01	67.0	0.0	0.3	0.0	0.0
	r02	57.6	0.2	0.5	0.2	0.1
	r03	54.8	1.2	1.6	1.0	1.2
	r04	69.7	0.2	0.4	0.2	0.7
	r05	5.0	0.1	0.2	0.1	0.1
	r06	63.9	0.1	0.1	0.1	0.1
	r07	50.0	0.4	0.5	0.4	0.7
	r08	44.4	0.0	0.1	0.0	0.0
	r09	58.9	0.2	0.3	0.2	0.2
	r10	78.5	0.1	0.2	0.1	0.2
	r11	38.8	0.2	0.1	0.1	0.2
	r12	1.4	0.0	0.1	0.0	0.0
**Pregnancy**	r01	60.2	0.0	0.0	0.0	0.1
	r02	54.6	0.1	0.1	0.0	0.1
	r03	50.2	0.1	0.2	0.1	0.2
	r04	88.5	0.0	0.0	0.0	0.0
	r05	70.3	0.1	0.0	0.0	0.1
	r06	62.8	0.1	0.1	0.0	0.3
	r07	67.7	0.2	0.1	0.1	0.2
	r08	60.0	0.1	0.1	0.0	0.2
	r09	58.5	0.1	0.1	0.0	0.2
	r10	59.2	0.2	0.2	0.2	0.2

Variation coefficient (%) of SE for Labor and Pregnancy dataset.

**Table 9:** Variation coefficient (%) of PPV for Labor and Pregnancy dataset.

Dataset	Rec.	ABC	GWO	MFO	PSO	WOA
**Labor**	r01	42.6	0.0	0.2	0.0	0.0
	r02	37.9	0.1	0.4	0.2	0.2
	r03	21.4	1.3	1.8	1.1	1.2
	r04	41.6	0.3	0.5	0.3	0.9
	r05	5.9	0.1	0.2	0.1	0.1
	r06	40.9	0.1	0.1	0.1	0.1
	r07	32.9	0.5	0.5	0.4	0.7
	r08	32.7	0.0	0.1	0.0	0.0
	r09	38.8	0.3	0.4	0.2	0.2
	r10	49.8	0.1	0.2	0.1	0.2
	r11	28.6	0.2	0.1	0.1	0.2
	r12	1.6	0.0	0.1	0.0	0.0
**Pregnancy**	r01	24.0	0.1	0.1	0.0	0.1
	r02	34.1	0.1	0.1	0.0	0.1
	r03	34.1	0.1	0.1	0.1	0.1
	r04	47.4	0.0	0.0	0.0	0.0
	r05	37.5	0.1	0.1	0.0	0.1
	r06	35.5	0.1	0.1	0.1	0.4
	r07	33.4	0.2	0.2	0.1	0.3
	r08	30.2	0.1	0.2	0.1	0.3
	r09	10.1	0.1	0.1	0.1	0.2
	r10	33.8	0.2	0.2	0.1	0.2

Variation coefficient (%) of PPV for Labor and Pregnancy dataset.

**Table 10:** Variation coefficient (%) of F1 for Labor and Pregnancy dataset.

Dataset	Rec.	ABC	GWO	MFO	PSO	WOA
**Labor**	r01	60.1	0.0	0.3	0.0	0.0
	r02	52.4	0.2	0.4	0.2	0.2
	r03	44.9	1.3	1.7	1.0	1.2
	r04	61.2	0.2	0.4	0.3	0.8
	r05	5.4	0.1	0.2	0.1	0.1
	r06	58.3	0.1	0.1	0.1	0.1
	r07	45.5	0.5	0.5	0.4	0.7
	r08	41.4	0.0	0.1	0.0	0.0
	r09	53.9	0.2	0.3	0.2	0.2
	r10	70.5	0.1	0.2	0.1	0.2
	r11	35.8	0.2	0.1	0.1	0.2
	r12	1.5	0.0	0.1	0.0	0.0
**Pregnancy**	r01	47.3	0.0	0.1	0.0	0.1
	r02	48.8	0.1	0.1	0.0	0.1
	r03	45.0	0.1	0.1	0.1	0.1
	r04	78.9	0.0	0.0	0.0	0.0
	r05	58.9	0.1	0.0	0.0	0.1
	r06	55.2	0.1	0.1	0.0	0.3
	r07	58.2	0.2	0.1	0.1	0.2
	r08	49.9	0.1	0.2	0.1	0.2
	r09	40.2	0.1	0.1	0.1	0.2
	r10	49.4	0.2	0.2	0.2	0.2

Variation coefficient (%) of F1 for Labor and Pregnancy dataset.

In the next step, we focused on comparing the medians of the extraction accuracy for the GWO, MFO, PSO, and WOA algorithms. Due to the very low variability of the ACC in the repeated experiments for individual records and algorithms that can be considered comparable with respect to the variability of the measurements, the ACCs in the repeated experiments were approximated by their median for further analysis, and then Friedman's test was applied. According to the results of this test, the differences in medians of the ACC for the GWO, MFO, PSO, and WOA algorithms cannot be considered statistically significant for neither the Labor database (χ^2^(3) = 0.138, *p* = 0.987) or the Pregnancy database χ^2^(3) = 5.09, *p* = 0.165.

Kendall's W indicated a negligible effect size for both the Labor and Pregnancy datasets, suggesting that the variability in median ACC values attributable to the choice among GWO, MFO, PSO, and WOA was minimal (Labor dataset: Kendall's W = 0.004, Pregnancy dataset: Kendall's W = 0.170). Therefore, although small numerical differences between algorithms were observed, these differences cannot be considered practically meaningful. This interpretation is consistent with the pairwise Cohen's d values, which were close to zero for comparisons among GWO, MFO, PSO, and WOA.

Pairwise effect sizes based on paired Cohen's d further supported the observed differences between the algorithms. For the Labor dataset, comparisons between the ABC algorithm and the remaining algorithms (GWO, MFO, PSO, and WOA) yielded absolute Cohen's d values greater than 2, indicating extremely large practical differences in extraction accuracy. In contrast, comparisons among GWO, MFO, PSO, and WOA produced Cohen's d values close to zero, suggesting negligible practical differences between these algorithms.

An even stronger effect was observed for the Pregnancy dataset, where the absolute values of Cohen's d for comparisons between ABC and the remaining algorithms exceeded 5. This indicates an exceptionally large practical difference in favor of GWO, MFO, PSO, and WOA. Similarly to the Labor dataset, pairwise comparisons among these four algorithms resulted in effect sizes close to zero, confirming that their extraction performance was practically equivalent.

These findings demonstrate that the inferior performance of the ABC algorithm was not only statistically significant but also highly relevant from a practical perspective.

## 6 Discussion

The experiments presented in this paper showed that the combination of SA with metaheuristic methods was able to effectively extract fECG in most cases. However, in some cases, the quality of the extraction was not sufficient and therefore we present factors that can affect the quality of the extraction and thus the performance of the algorithm. The factors selected for further analysis and discussion were as follows: (1) parameter settings, (2) input signals' quality, (3) electrode combination selected, and (4) time complexity of the algorithm.

**Figure 2:**
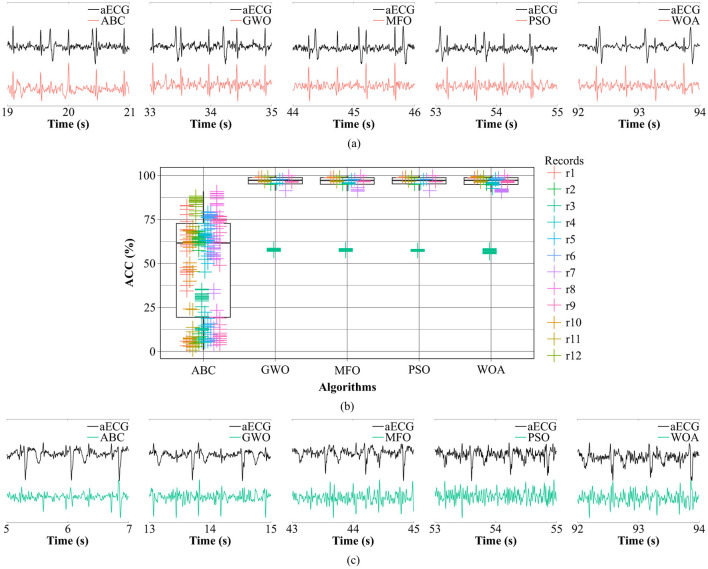
Visual representation of the results from Labor dataset. **(a)** Examples of the input and output signals for r1 record, **(b)** hybrid boxplots for all records from Labor dataset, and **(c)** examples of the input and output signals for r3 record.

**Figure 3:**
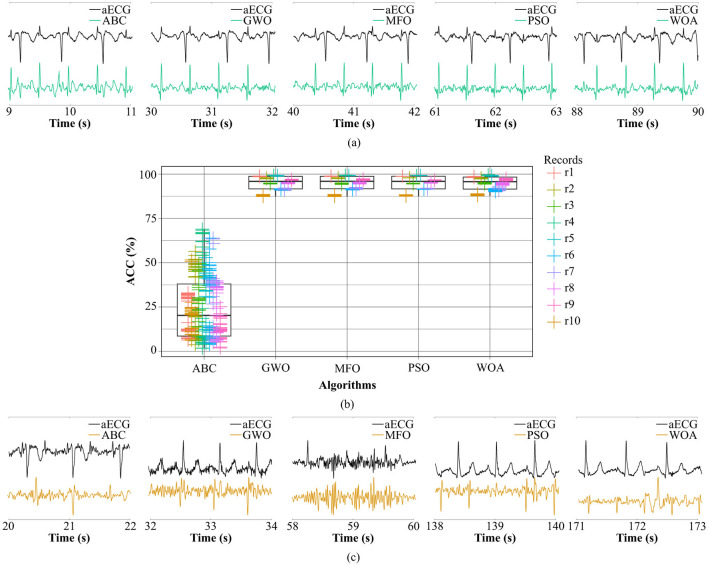
Visual representation of the results from Pregnancy dataset. **(a)** Examples of the input and output signals for r4 record, **(b)** hybrid boxplots for all records from Pregnancy dataset, and **(c)** examples of the input and output signals for r10 record.

The parameters of the algorithms themselves are a key factor to keep in mind. After some preliminary experiments, the two parameters: *population size* and *maximum iteration* were set to 10 and 10, respectively. It was followed up with another experiment with 20 and 20 as the population size and maximum iteration, respectively. In this article, we show the results of the second parameter setting and the result of the second setting can be observed in Figures 2, Figure 3. The decision to display only one set of results, specifically those from the second parameter setting, was driven by the extensive nature of the findings. Both sets of results were similar, and to avoid redundancy in the article, we opted to showcase a representative set. By doing so, we aimed to streamline the presentation of our research, ensuring that the article remains focused and easily digestible for the readers. This selective approach allows us to highlight the key outcomes without overwhelming the audience with redundant information.

The hybrid boxplots in Figures 2, 3 show that the ABC algorithm produced substantially lower and more variable ACC values than the remaining algorithms. In contrast, GWO, MFO, PSO, and WOA exhibited narrow distributions concentrated around high ACC values for most records, indicating stable and robust extraction performance across repeated runs. The main exceptions were Labor record r03 and Pregnancy record r10, where all algorithms achieved lower ACC values. This suggests that the quality of the input aECG signal represented a limiting factor independent of the optimization algorithm used. These graphical observations are consistent with the reported effect sizes, which demonstrated large practical differences between ABC and the remaining algorithms, but negligible differences among GWO, MFO, PSO, and WOA.

Another factor that heavily influences the result is the input that is fed to the algorithm. This fact is illustrated by Figures 2, 3 for records from the Labor and Pregnancy dataset, respectively. In both cases, we present examples of input aECG signals of different quality (high and low) along with the extracted signals by individual algorithms. We also include hybrid boxplots showing ACC values separately for each record. Figure 2a illustrates the extraction performed by individual algorithms using a high-quality input aECG signal from record r1. In this scenario, the signal is not burdened with a high level of interference, and the fetal component is acquired with sufficient amplitude relative to the maternal one. As a result, all algorithms, including ABC, achieved effective mECG suppression and high-quality fECG extraction. On the contrary, Figure 2c depicts the use of a low-quality input aECG signal from record r3, which is loaded with a high level of interference. Unfortunately, it was not possible to extract a high-quality fECG using any of the tested algorithms in this case. Consequently, all algorithms yielded low ACC values for this recording, as evident in sample Figure 2b.

In Figure 3, we also analyzed two examples of high and low quality aECG signals using r4 and r10 records, respectively, this time for Pregnancy dataset. Most tested algorithms successfully extracted the resulting fECG, except for ABC. ABC's failure to extract the fECG might be attributed to a very prominent maternal component and a lower level of the fetal component in these signals. On the other hand, in example Figure 3c, we utilized recording r10, which had a very low level of fECG in the input signal and was also burdened with interference. For this recording, all algorithms demonstrated inefficient extraction. The accurate detection of fetal R-peaks was not possible, resulting in significantly lower ACC values compared to other recordings, as indicated in the hybrid boxplots in Figure 3b.

The area of signal quality assessment in the field of fECG monitoring does not have standard metrics the clinicians and researchers agree upon (40, 41). The quality of the input aECG signal is affected by various external factors of a biological and technical nature. These factors include, for example, maternal body composition and signals produced by it (especially mECG), fetal position, motion artifacts, noise generated by electronic devices or power-line, skin and electrode properties and so on. The more of these factors are in play, the more difficult the extraction becomes, therefore one must keep in mind that acquisition of a high-quality aECG input is the first step to extracting precise fECG signal and thus obtaining the necessary clinical information. The measurement conducted results in 4 aECG signals. The various combinations of these 4 signals result in different extractions. To give a fair review of the algorithm one set of electrode combinations was chosen for each record. When selecting the best outcome for each algorithm, the electrode combination varies.

Another factor related to the algorithms is the time complexity of the algorithm. The convergence time depends on the parameters such as the iteration count and the population size. For this study, different parameter settings were tested to observe a significant change in accuracy, but it did not result in a better extraction. In contrast to a non-adaptive SA algorithm that does not adapt to each maternal complex, but takes an average of all the complexes to create a maternal template; the optimization algorithm adapts to each maternal component detected, which also raises the computational cost.

To quantitatively assess this computational complexity, we measured the runtime (in seconds) of the compared population-based algorithms for various population sizes (*N*) and maximum numbers of iterations (*iter*), as presented in Table 11. As previously mentioned, all evaluations were performed in MATLAB R2023a on a workstation equipped with an Intel Core i9-10980XE processor, an NVIDIA GeForce GTX 1050 Ti GPU, and 64 GB of RAM. The table reports the median execution times obtained from 30 independent runs.

**Table 11:** Median of time requirements (in seconds) for different population sizes (N) and maximum iterations (iter) across the compared bio-inspired algorithms for the Labor (150,000 samples) and Pregnancy datasets (600,000 samples).

Dataset	Rec.	***N*** = 10, ***iter*** = 10					***N*** = 20, ***iter*** = 20				
		**ABC**	**GWO**	**MFO**	**PSO**	**WOA**	**ABC**	**GWO**	**MFO**	**PSO**	**WOA**
**Labor**	r1	221.32	214.07	214.66	228.13	214.35	874.91	850.01	851.30	892.66	850.48
	r2	164.75	160.26	160.75	166.06	159.95	652.88	632.88	633.08	653.56	633.28
	r3	167.15	162.77	163.24	172.54	162.43	663.42	643.73	643.39	676.43	645.46
	r4	151.46	146.51	146.85	152.15	146.58	596.85	578.93	579.56	596.76	580.24
	r5	215.89	209.08	210.38	217.63	208.88	852.63	825.10	829.10	849.35	827.30
	r6	218.75	213.3	213.98	225.92	212.55	865.55	840.59	839.25	879.16	838.71
	r7	149.86	145.55	146.04	154.27	145.28	592.15	574.82	574.60	603.64	574.13
	r8	208.85	202.59	203.44	215.39	202.63	826.23	802.36	801.54	841.31	800.43
	r9	152.82	148.56	148.6	157.21	147.91	606.34	587.95	586.59	615.73	586.41
	r10	208.19	200.9	201.33	213.95	200.68	820.84	796.94	796.91	836.19	797.81
	r11	141.05	136.59	136.69	142.27	135.69	558.40	540.92	540.83	556.21	539.64
	r12	139.53	135.54	135.83	144.03	134.92	553.50	537.01	536.83	562.69	536.51
**Pregnancy**	r1	2,049.23	1,980.2	1,982.19	2,063.18	1,968.43	8,041.41	7,716.39	7,797.32	8,038.55	7,715.95
	r2	1,577.71	1,509.35	1,504.37	1,562.6	1,510.22	6,146.07	5,949.00	5,971.12	6,171.14	5,945.57
	r3	2,233.86	2,144.59	2,127.78	2,274.74	2,128	8,710.48	8,382.24	8,454.26	8,753.12	8,391.29
	r4	1,402.87	1,339.43	1,332.1	1,383.09	1,326.61	5,441.40	5,260.93	5,294.42	5,449.44	5,272.96
	r5	1,462.34	1,400.42	1,389.87	1,457.44	1,385.06	5,696.05	5,495.49	5,540.05	5,704.35	5,474.14
	r6	1,208.9	1,160.11	1,164.68	1,217.74	1,163.09	4,735.80	4,559.38	4,592.63	4,747.40	4,584.96
	r7	1,339.63	1,279.84	1,277.59	1,334.46	1,269.86	5,217.21	5,055.93	5,089.98	5,195.93	5,036.10
	r8	1,186.93	1,140.32	1,142.8	1,189.18	1,144.4	4,642.78	4,478.05	4,493.19	4,615.80	4,475.32
	r9	3,030.49	2,905.53	2,878.08	3,021.76	2,862.79	11,728.11	11,346.75	11,419.24	11,714.00	11,349.65
	r10	2,549.87	2,453.22	2,457.76	2,587.89	2,454.31	9,970.62	9,550.18	9,684.08	10,002.80	9,607.12

Median of time requirements (in seconds) for different population sizes (N) and maximum iterations (iter) across the compared bio-inspired algorithms for the Labor (150,000 samples) and Pregnancy datasets (600,000 samples).

The Labor dataset records contain 150,000 samples, whereas the Pregnancy dataset records consist of 600,000 samples. Although the signal length increased by four, the computation time increased ten times, indicating a non-linear scaling of computational cost with respect to the data length. This trend was consistent across all tested parameter settings. Furthermore, while the overall execution times did not differ drastically among the tested algorithms, a discernible trend was observed: ABC and PSO were the most computationally demanding, exhibiting similar runtimes, whereas the remaining three methods (GWO, MFO, and WOA) were faster and demonstrated comparable computational efficiency.

Regarding clinical applicability, the current computational demands render the deployment of these algorithms for strict, beat-to-beat real-time fetal monitoring unfeasible. However, the achieved processing times are highly appropriate for offline diagnostic evaluations, such as the detailed analysis of long-term ambulatory or home-monitoring recordings. It is important to emphasize that in this study, the population-based algorithms, combined with the SA method, were applied to the entire recordings at once. The main reason was to demonstrate the robustness of the proposed solution, which is critical for accurately determining the fetal condition. Given the non-linear scaling discussed above, analyzing shorter signal segments would drastically reduce the computational burden. Consequently, if the data were processed in short, sliding windows (e.g., a few seconds in length), the proposed framework could potentially be implemented in clinical applications with only a minimal processing delay. Future study could therefore focus on testing shorter segments and determining the minimum amount of data required for accurate fECG signal extraction, along with HRV analysis.

Finally, we examined the performance of the evaluated algorithms. No statistically significant differences were found among GWO, MFO, PSO, and WOA. In contrast, ABC showed markedly inferior performance in the SA-based fECG extraction task and was also substantially less stable across repeated runs. This behavior suggests that the standard ABC search mechanism was not well suited to the present optimization problem. A possible explanation is that the task is low-dimensional but highly sensitive to small changes in the estimated scaling coefficients. In such a setting, the relatively stochastic search behavior of standard ABC may be less suitable than methods that enable more consistent fine adjustment of a small number of parameters. As a result, even small errors in coefficient estimation may reduce the quality of mECG suppression and, consequently, subsequent fetal QRS detection. Similar limitations of ABC, including premature convergence, slow convergence, and a tendency to become trapped in local optima, have also been reported in previous studies (37–39). One way that was suggested by several authors was to improve on the already existing algorithm by either using a modified ABC (37) or combining it with PSO or other population-based algorithms (42, 43). To keep the comparison fair, we did not opt for a modified version of ABC as we used the original method for the rest of the algorithms.

Future research may therefore focus on the use of modified or hybrid variants of the optimization methods and their potential impact on extraction performance. In addition, the present study is limited by the relatively small size and limited diversity of the utilized datasets, as well as the absence of external validation. Future work should therefore also include evaluation on larger and more diverse NI-fECG datasets acquired under different clinical conditions and using different acquisition systems. In particular, the inclusion of independent external datasets would enable a more comprehensive assessment of the generalizability of the presented findings.

Recent studies (44–46) have also increasingly explored deep learning-based approaches for NI-fECG extraction and fetal QRS detection, which represent another important direction in this field. Although such methods were not included in the present controlled comparison, mainly because they differ substantially in terms of data requirements, training procedures, preprocessing strategies, and evaluation protocols, their growing relevance should be acknowledged when interpreting the scope of the present study. Future research should therefore consider not only systematic comparisons between optimization-based and deep learning-based approaches under unified evaluation conditions, but also the potential development of hybrid frameworks combining both methodological families. Such approaches could exploit the adaptability of optimization methods together with the representation-learning capability of deep learning models.

## 7 Conclusion

This study compared five population-based optimization methods within the same SA-based NI-fECG extraction pipeline. The results showed that GWO, MFO, PSO, and WOA achieved comparable extraction accuracy, whereas the ABC-based approach performed substantially worse. These findings suggest that, for this specific NI-fECG extraction task, several evaluated optimization methods provide similar outcomes, while others appear less suitable in terms of robustness and reliability, although these results should be interpreted with caution given the limited size and homogeneity of the datasets.

Certain factors influenced the extraction quality and algorithm performance. Among the most significant ones are: (1) parameter settings, such as population size and maximum iteration, (2) quality of the input signals, and (3) the choice of electrode combinations. Therefore, fine-tuning the parameters and ensuring high-quality input signals are crucial for optimal results. Future research should consequently focus on refining these aspects to unlock the full potential of this combined approach and pave the way for broader applications in clinical settings and beyond, while acknowledging that further validation, including real-time implementation and evaluation on larger and more diverse datasets, is necessary before clinical deployment. This can be achieved, for example, by standardizing electrode placements, automating the selection of measuring electrodes based on the signal quality and optimizing the extraction system's time complexity. A limitation of this study is the use of relatively small and homogeneous datasets without external validation; future work should therefore extend the evaluation to larger, more diverse, and independent NI-fECG datasets, as well as include broader benchmarking across different extraction approaches and evaluation metrics to strengthen the generalisability of the findings.

## Data Availability

The original contributions presented in the study are included in the article/supplementary material, further inquiries can be directed to the corresponding author.
